# The time-course of broiler intestinal microbiota development after administration of cecal contents to incubating eggs

**DOI:** 10.7717/peerj.3587

**Published:** 2017-07-20

**Authors:** Erin E. Donaldson, Dragana Stanley, Robert J. Hughes, Robert J. Moore

**Affiliations:** 1 Institute for Future Farming Systems, Central Queensland University, Rockhampton, Queensland, Australia; 2 Poultry Cooperative Research Centre, University of New England, Armidale, New South Wales, Australia; 3 Pig and Poultry Production Institute, South Australian Research and Development Institute, Roseworthy, South Australia, Australia; 4 School of Animal and Veterinary Sciences, The University of Adelaide, Roseworthy, South Australia, Australia; 5 School of Science, RMIT Univeritsy, Bundoora, Victoria, Australia

**Keywords:** Diet, Microbiota, Cecum, Gut, Chicken

## Abstract

**Background:**

The microbial populations that inhabit the gastrointestinal tract (GIT) are known to influence the health and growth performance of the host. Clean hatcheries and machine-based incubation practices in the commercial poultry industry can lead to the acquisition of aberrant microbiota in the GIT of chickens and a very high level of bird-to-bird variation. The lack of microbial profile flock uniformity presents challenges for harnessing and manipulating intestinal bacteria to better serve the host.

**Methods:**

Cecal contents from high or low performing chickens were used to inoculate the surface of eggs prior to hatching and then the initial gut colonisation was monitored and subsequent changes in gut microbiota composition were followed over time. Two different cecal treatment groups were compared to an untreated control group (*n* = 32). Bacterial communities were characterised using high-throughput 16S rRNA gene sequencing techniques.

**Results:**

Cecal microbiota transfer via egg surface application did not transfer the performance profile of the donors to the recipient birds. One of the cecal inoculations provided a more uniform gut microbiota, but this was not reproduced in the second group with a different inoculum. Development of the intestinal community was reproducible in all three groups with some genera like *Lactobacillus* showing no change, others like *Faecalibacterium* increased in abundance slowly and steadily over time and others like *Enterobacter* were abundant only in the first days of life.

**Discussion:**

The cecal treatment reduced bird-to-bird variation in microbiota composition. Although the high FCR performance of donor birds was not transferred with the cecal microbiota, all three groups, including the control, performed better than standard for the breed. The pattern of microbiota development was similar in all three flocks, indicating that the normal processes of microbiota acquisition largely swamped any effect of the cecal material applied to eggs.

## Introduction

It is well recognised that the establishment of the gastrointestinal tract microbiota commences from birth (Leser & Molbak, 2009). In human infants, microbiota have a high diversity by the age of one year. It is estimated that the human microbiota composition fluctuates and develops up until the age of four years when it is considered to be fully matured. In the poultry industry, studies indicate that the timeframe to maturity of broiler chickens is significantly reduced and it appears that microbiota can stabilise within three days post-hatch (Apajalahti, Kettunen & Graham, 2016) and remain reasonably constant until 30 days of age (Lu et al., 2003). Maturation of microbiota assumes a stable environment with the ability to resist change (Bonetti, 2002; Engelbrektson et al., 2006). Colonisation of the chicken GIT is thought to start immediately after hatch and therefore the hatching environment can have a major influence on the microbial profile. Large differences in microbiota profiles have been reported in the chicken (Stanley et al., 2013c). This kind of variation has also been found in the GIT microbiota of humans and other animals, and is attributed to both host and environmental factors (Durso et al., 2010; Lagier et al., 2012).

Amongst the animal production systems, broiler chickens are notably different, in that the parents play no part in incubation or rearing of the young. This separation markedly reduces parental influence on the development of microbiota. Furthermore, the implementation of strict hygiene practices by commercial hatcheries reduces bacterial load in the hatching environment and limits the spread of bacterial pathogens. Therefore, newly hatched chicks are exposed to a diverse range of bacteria from environmental sources rather than from parental sources. After hatch, chicks are exposed to various microbes in the environments in the hatchery, during transport, and on arrival at the farm (Stanley et al., 2013c).

The diversity in bacterial sources in combination with the lack of parentally derived bacteria during the first hours and days of life is suggested to be the reason for the widely varying colonisation of the chicken GIT (Fuller, 1989; Stanley et al., 2013c). This situation is somewhat analogous to the findings that human infants delivered in hospitals can harbour aberrant microbiota rather than human specialised microbiota (Schwiertz et al., 2003) and infants delivered by caesarean section have been shown to have different microbiota compared to naturally delivered infants (Dominguez-Bello et al., 2010). However, a key difference is the physical and permanent separation of chicken eggs from hens on breeder farms. Once the eggs have been washed or fumigated prior to hatching, there is no further contact with adult chickens during incubation or immediately post-hatch (Varmuzova et al., 2016).

The extensive inter-flock and intra-flock variation that occurs in the intestinal microbiota composition of birds may result in different bacterial metabolites and products establishing different body chemistry. This in turn will influence the bird response to feeding, probiotics and prebiotics, and, potentially, any administered medicine. It is suggested that the initial inoculation and colonisation of the chicken GIT microbiota can have a major influence on the growth performance and health of birds (Guarner & Malagelada, 2003; Sears, 2005) as well as flock microbiota uniformity and reproducibility (Stanley et al., 2013c). We endeavoured to stabilise the initial colonisation of the GIT of broiler chickens by the application of cecal microbiota from extreme performance birds onto eggs in the day before hatch. Our hypothesis was that this seeded microbiota would thus be the first bacteria encountered by the newly hatched chicks; a uniform initial exposure may led to more uniform mature microbiota structure across the exposed birds. We investigated the influence of this intervention on development of cecal microbiota over time and intra- and inter-flock differences.

## Materials & Methods

### Animal trials

Two identical trials were conducted eight weeks apart. For each of the trials, Cobb 500 fertile eggs (total 150) were obtained from the Baiada Hatchery (Willaston, South Australia) from the same breeder stock, and incubated for 18 days in a single incubator (IM Incubators model IM288; 38 °C, 55% relative humidity, turning hourly). Fertile eggs were allocated by weight into three inoculation treatment groups and transferred to three separate incubators (Intensive Farming Supplies, Cavan, SA 5094, model MPS24 A; 36.7 °C and 66% relative humidity). On the day before hatching, 12–15 eggs at a time were removed from the incubators, wiped with 70% ethanol then swabbed with (1) control PBS solution, (2) cecal contents inoculum 1, diluted in PBS, and (3) cecal contents inoculum 2, diluted in PBS.

The inocula for both cecal content transfer trials were selected from our previous trial (Crisol-Martinez et al., 2017). The donors were selected from four birds with relatively high FCR for inoculum 1, and four birds with low FCR for inoculum 2. The cecal samples selected for use in the inocula were also selected based on the previously determined microbiota composition with the cecal samples used in inoculum 2 having particularly high levels of *Bacteroides*. Thus the two inocula used were derived from birds with different FCR performance and distinctly different micorbiotas. Each inoculum was prepared by mixing equal weights cecal content from each of four birds and diluting in sterile PBS. The inocula (0.3 ml) were painted onto the upper half of the shells (blunt end up) of each egg using a cotton bud. Eggs were returned to the incubator after less than 10 min.

Newly hatched chickens were weighed individually then transferred in inoculation treatment groups to three separate floor pens in a temperature controlled room (day 0). There was no difference in mortality or hatchability between the three groups. There was physical separation between treatment groups at all times and staff wore disposable gloves when handling chickens, feeders and drinkers. All chickens received a proprietary diet formulated for this breed comprised of 44.4% wheat, 17% soybean meal, 15% barley, 10% canola meal, 5% peas, 3.2% meat meal, 3% tallow, 1% limestone, 0.5% vitamin mix, and traces of salt, lysine HCl, DL-methionine and threonine. The feed was antibiotic and ionophore free. There was no specific coccidiosis control method used. The same batch of commercially prepared starter crumbles (Ridley Agriproducts, Murray Bridge, South Australia) was used in both trials and was stored under controlled cool and dry conditions for the duration of the trials. Birds were fed ad libitum and had access to water from drinking nipples at all times. Birds had 23 h of light for the first three days and then 12 h of light for the remainder of the experimental period.

At 15 days of age, the birds were transferred in pairs to metabolism cages in a temperature controlled room. The initial pairing was done to minimise stress and allow the birds to adjust to cages. At day 17, birds were moved into individual metabolism cages. Individual caging allowed the precise assessment of individual feed intake, energy in feed, and unused energy remaining in feces. The experimental design eliminated competition for feed and reduced behavioural issues affecting feed intake. Single bird caging and individual measurements and sampling were implemented in order to allow direct correlation of microbiota structure and productivity measurements on a bird by bird basis.

The apparent metabolisable energy (AME) values of the commercial broiler diet were determined in a classical 7-day AME study involving measurements of total feed intake and total excreta output and subsequent measurement of gross energy (GE) values of feed and excreta by isoperiobol bomb calorimetry. AME in MJ/kg dry matter, was calculated as (AMEdiet  = [(GEdiet × feed eaten) × (GEexcreta × dry excreta)]/feed eaten/dry diet content) (Stanley et al., 2013a).

Feed intake (FI) was measured during the adaptation and collection phases of the study. All excreta were collected daily during the 4-day collection phase and dried overnight at 90 °C. Birds were weighed at the start and end of the 7-day period then retained in the cages until 27 days of age. Feed consumption was measured by weighing the feed at the start (day 17) and end (day 24) of the AME period. On day 24, feed was weighed into hoppers and the residue weighed on day 27 in order to provide feed intake and feed conversion ratio data up until the time the experiment was terminated. All of the above measurements were taken from day 17 to day 24, during the time when single birds were housed in metabolic cages. The performance analysis was completed on both trials separately, however sequencing was performed using the samples from the trial 1. Fecal samples (via cloacal swab) were collected at multiple time points from day 0 to day 23 and frozen immediately for DNA extraction. Although all birds were sampled at each time-point, due to lower fecal content some swabs yielded too little DNA to be sequenced. A total of 160 samples were successfully sequenced in this study. All 96 birds were killed by cervical dislocation. The sex of each chicken was determined by visual observation of gonads when dissected.

### DNA preparation, sequencing, and data analysis

DNA was prepared, sequenced and analysed as detailed by Stanley et al. (2013a). The primers used to PCR amplify the V1–V3 region of the 16S rRNA gene were (forward primer, 5′ AGAGTTTGATCCTGG 3′; reverse primer, 5′ TTACCGCGGCTGCT 3′). Sequencing was performed using a Roche/454 FLX+ Genome Sequencer and Titanium chemistry. Sequences were analysed using QIIME (Caporaso et al., 2010), denoised and error corrected with Acacia (Bragg et al., 2012) and chimera checked using Pintail (Ashelford et al., 2005). The sequences were then quality trimmed with length from 300 to 600 bases, no ambiguous nucleotides and a maximum of 6 nucleotides in homopolymer runs. OTU picking was done at 3% divergence level using the UCLUST (Edgar, 2010) algorithm. OTUs represented with less than 10 sequences and present in less than 5 samples were filtered out of the analysis. QIIME generated abundance table based data were further analysed and visualised using Primer-E (http://www.primer-e.com/) and Calypso (Zakrzewski et al., 2017). The sequencing data is publicly available on MG-RAST database under library number mgl564762 and a project ID mgp21821.

### Statistical analysis

SAS for Windows version 9.4 software package (SAS Institute Inc., Cary, NC, USA) was used to determine whether data were normally distributed (Univariate procedure; Shapiro–Wilk test), then two-way analysis of variance with the General Linear Model (GLM) procedure was used to examine the factors inoculation treatment and sex of chicken, and the interaction between treatment and sex. Significant differences between treatments and sex were determined by Duncan’s Multiple Range Test. Microbiota statistical analysis was done in Calypso (Zakrzewski et al., 2017) for Richness, Evenness, ANOSIM, ANOVA, network, correlations and LEfSe. UniFrac matrixes were calculated in QIIME (Caporaso et al., 2010) and PERMANOVA was done in Primer-E (http://www.primer-e.com/).

### Animal ethics statement

Animal ethics approvals were obtained from the University of Adelaide (S-2013-149) and Primary Industries and Regions South Australia (14/13).

## Results

### Live weight, feed intake, feed conversion and metabolisable energy

The effects of inoculation treatments and sex of chicken on live weight, feed intake, feed conversion ratio and apparent metabolisable energy of the diet are summarised in Tables 1 and 2. Inoculation treatment 1 significantly reduced live weight at 15 and 22 days of age compared with the control, but had no effect on feed intake, feed conversion, or apparent metabolisable energy value of the diet in the first experiment (Table 1). In contrast, in the second experiment, inoculation treatments had no effect on live weight, feed intake, and feed conversion, but appeared to have a small but significant effect on apparent metabolisable energy with inoculant 2 depressing AME value by 1.6% for male chickens only (Table 2). In both experiments, male chickens were heavier, ate more feed, and converted more efficiently than female chickens, but there were no significant differences in AME values due to gender alone.

**Table 1:** Trial 1—Effects of inoculation treatments and sex of chicken on live weight at the start of the 7-day metabolism study (BW15, in g/bird), live weight at the end (BW22, in g/bird), feed intake (FI, in g/bird), feed conversion ratio (g feed: g gain) and apparent metabolisable energy of the diet (AME, in MJ/kg dry matter basis).

**Summary of analysis of variance**					
	BW15	BW22	FI	FCR	AME
Treatment (T)	^***^	^**^	ns	ns	ns
Sex (S)	^*^	^***^	^**^	^***^	ns
T × S	ns	ns	ns	ns	ns
Mean	621	1,149	741	1.412	15.38
CV	7.6	9.3	10.3	5.1	2.8

**Effect of inoculation treatments**						
Treatment	Chickens	BW15	BW22	FI	FCR	AME
Control	30	638 a	1,189 a	762 a	1.387 b	15.46 a
Inoculant #1	32	596 b	1,105 b	720 b	1.426 a	15.31 a
Inoculant #2	31	630 a	1,154 ab	742 ab	1.422 ab	15.37 a

**Effect of sex of chicken**						
Sex	Chickens	BW15	BW22	FI	FCR	AME
Female	48	613 b	1,115 b	720 b	1.440 a	15.42 a
Male	45	630 a	1,184 a	763 a	1.383 b	15.33 a

**Notes.**

* *P* < 0.05.

** *P* < 0.01.

*** *P* < 0.001.

ns *P* >0.05, CV is the coefficient of variation.

Means within the same column with a common letter are not significantly different *P* >0.05.

**Table 2:** Trial 2—Effects of inoculation treatments and sex of chicken on live weight at the start of the 7-day metabolism study (BW15, in g/bird), live weight at the end (BW22, in g/bird), feed intake (FI, in g/bird), feed conversion ratio (g feed: g gain) and apparent metabolisable energy of the diet (AME, in MJ/kg dry matter basis).

**Summary of analysis of variance**					
	BW15	BW22	FI	FCR	AME
Treatment (T)	ns	ns	ns	ns	ns
Sex (S)	^***^	^***^	^***^	^***^	ns
T × S	ns	ns	ns	ns	^*^
Mean	624	1,190	759	1.346	14.51
CV	9.2	8.7	9.6	4.3	1.7

**Effect of inoculation treatments**						
Treatment	Chickens	BW15	BW22	FI	FCR	AME
Control	32	612 a	1,176 a	755 ab	1.342 a	14.56 a
Inoculant #1	32	616 a	1,167 a	735 b	1.339 a	14.53 a
Inoculant #2	32	644 a	1,226 a	789 a	1.358 a	14.44 a

**Effect of sex of chicken**						
Sex	Chickens	BW15	BW22	FI	FCR	AME
Female	43	593 b	1,109 b	704 b	1.368 a	14.52 a
Male	53	649 a	1,256 a	804 a	1.329 b	14.50 a

**Effect of the interaction between treatment and sex on AME of the diet**						
Sex	Chickens	Control	Chickens	Inoculant #1	Chickens	Inoculant #2
Female		14.50 ab		14.51 ab		14.57 a
Male		14.60 a		14.55 a		14.35 b

**Notes.**

* *P* < 0.05.

** *P* < 0.01.

*** *P* < 0.001.

ns *P* > 0.05, CV is the coefficient of variation.

Means within the same column with a common letter are not significantly different *P* > 0.05.

Means with a common letter are not significantly different *P* > 0.05.

### Cecal transfer

**Figure 1:**
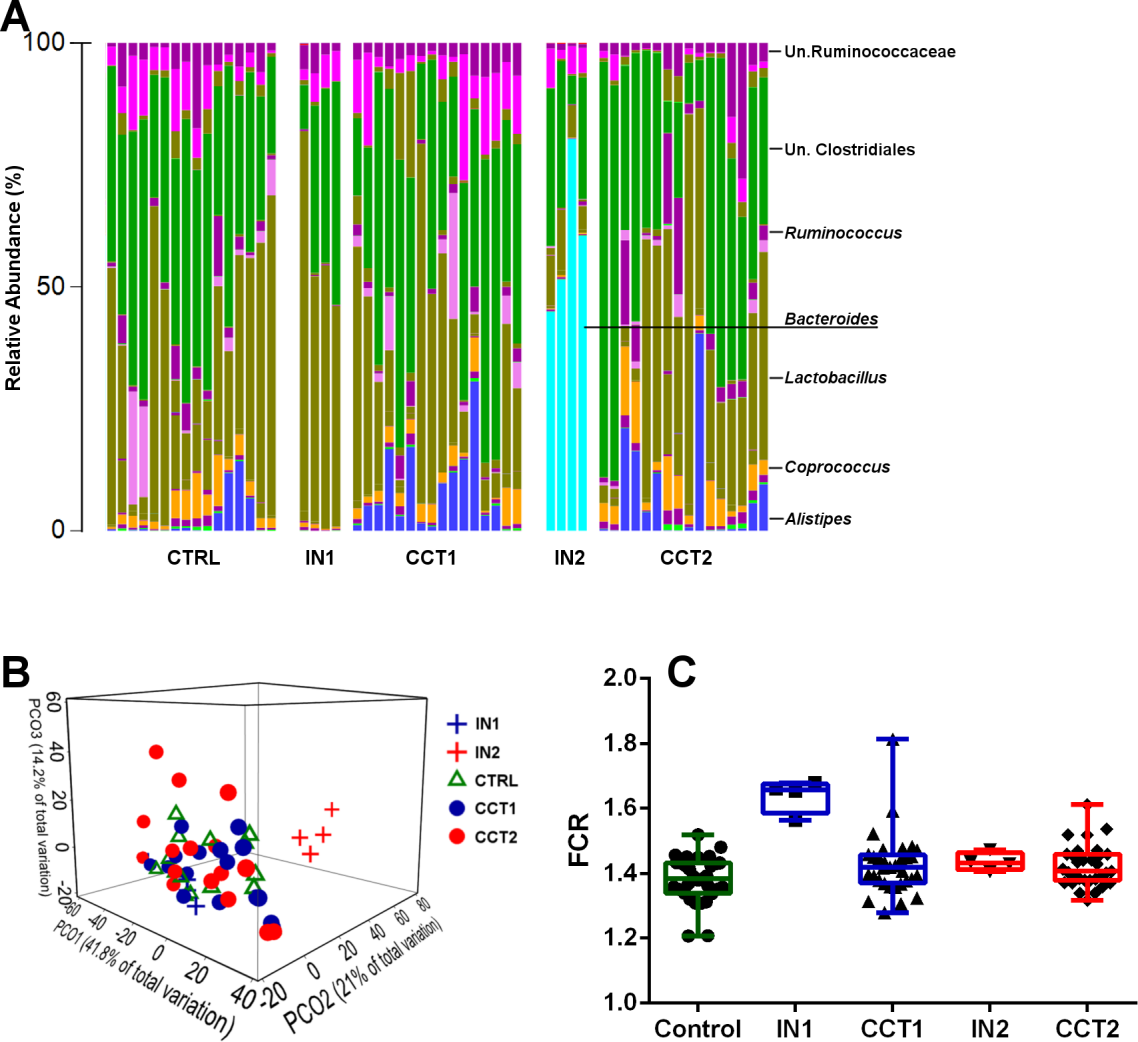
Barchart showing cecal microbiota at the genus level of all 3 treatment groups and their corresponding inoculums (A). Bray–Curtis genus level PCoA plot (B) and FCR differences between the groups and inoculums used for cecal content swabbing (C). CTRL = Control, IN1 = Donors for the inoculum used for Cecal Content Treatment 1 (CCT1); IN2 = Donors for the inoculum used for Cecal Content Treatment 2 (CCT2).

The genus level cecal microbiota profiles of control and the two cecal inocula treated bird groups, compared to their respective donor inocula are shown in Fig. 1A. The high performing bird derived inoculum 2 was dominated with *Bacteroides* that were absent in the birds of poor performance represented in inoculum 1. However, *Bacteroides*, an obligate anaerobic genus, was not transferred to the recipient birds. The presence of this dominant genus and its absence in other groups resulted in clear distinction of the microbiota in inoculum 2 group of donor birds, away from all other groups, including inoculum 1 (Fig. 1B). Based on microbiota profiles and the lack of significant difference in FCR between the control and either of inoculum treated groups (Fig. 1C), under the present conditions and with the current microbiota profiles used as inoculum, FCR-based performance was not affected in the recipient birds.

### Microbiota composition of cloacal swabs

The birds were sampled at multiple time points from day 0 to day 23. However, only very low levels of DNA could be isolated from the cloacal swabs on day 0 and most of the samples could not be PCR amplified and hence no sequence data was obtained. Therefore, only results from day 1 to day 23 are presented. 16S amplicons from 160 cloacal samples were sequenced and presented in the analysis. Although nine phyla were detected across the three treatment groups, there was a clear domination of Firmicutes in all three groups (Figs. S1, S2A). At a genus level *Lactobacillus* was the dominant genus (mean abundance of 40.39%), followed by *Enterococcus* (23.32%), *Clostridium* (3.7%), F*aecalibacterium* (2.27%), *Ruminococcus* (2.07%), *Staphylococcus* (1.77%), *Coprobacillus* (1.62%), *Coprococcus* (1.52%) and *Sphingomonas* (1.29%) with all other genera being of much lower abundance (<1%) (Fig. S1). There was a noticeable bird-to-bird variation in a few of the birds in each group (Fig. S1), for example, minimum abundance for the genus *Lactobacillus* was 0% (in a bird dominated by *Enterococcus* at 98.86%) while maximum of *Lactobacillus* abundance was 99.54%.

### Bird-to-bird microbiota variation

The high level of individual bird-to-bird variation in all three treatments implied that the issue of high intra flock variability was not remedied with cecal content treatments CCT1 and CCT2. To further investigate whether uniformity was increased using cecal content treatment ANOSIM was used to compare inter and intra group distances using Bray Curtis (Figs. S2B, S2D) and weighted and unweighted UniFrac at an OTU level (Figs. S2E, S2F). In all cases, the birds were most similar (or least dissimilar) in the CCT2 group; however there were no differences between the control and CCT1 treatment. All sample to sample distances within each group (bird-to-bird distances in the birds from the same group) were compared by weighted and unweighted UniFrac using ANOVA and Tukey Honestly Significant Differences test (using R). Sample to sample differences by weighted UniFrac were (ANOVA *P* = 1.165*E*^−4^) different, significantly between CCT1-CCT2 and CTRL-CCT2 (Fig. S2E). Similarly, using unweighted UniFrac the three groups had very different intra group distances (ANOVA, *P* = 2.2*E*^−16^), also significantly lower in CCT2 then in the other two groups and insignificant between CTRL and CCT1 (Fig. S2F). The above data indicates that CCT2 did achieve better uniformity and microbiota reproducibility; however this was not reproduced in CCT1 treatment and thus could depend on inoculum composition. In the development timeline, ANOSIM analysis showed that samples taken on day 1 were most dissimilar and samples taken on day 23 most similar (Fig. S2D) indicating less microbiota fluctuations in more mature birds.

**Figure 2:**
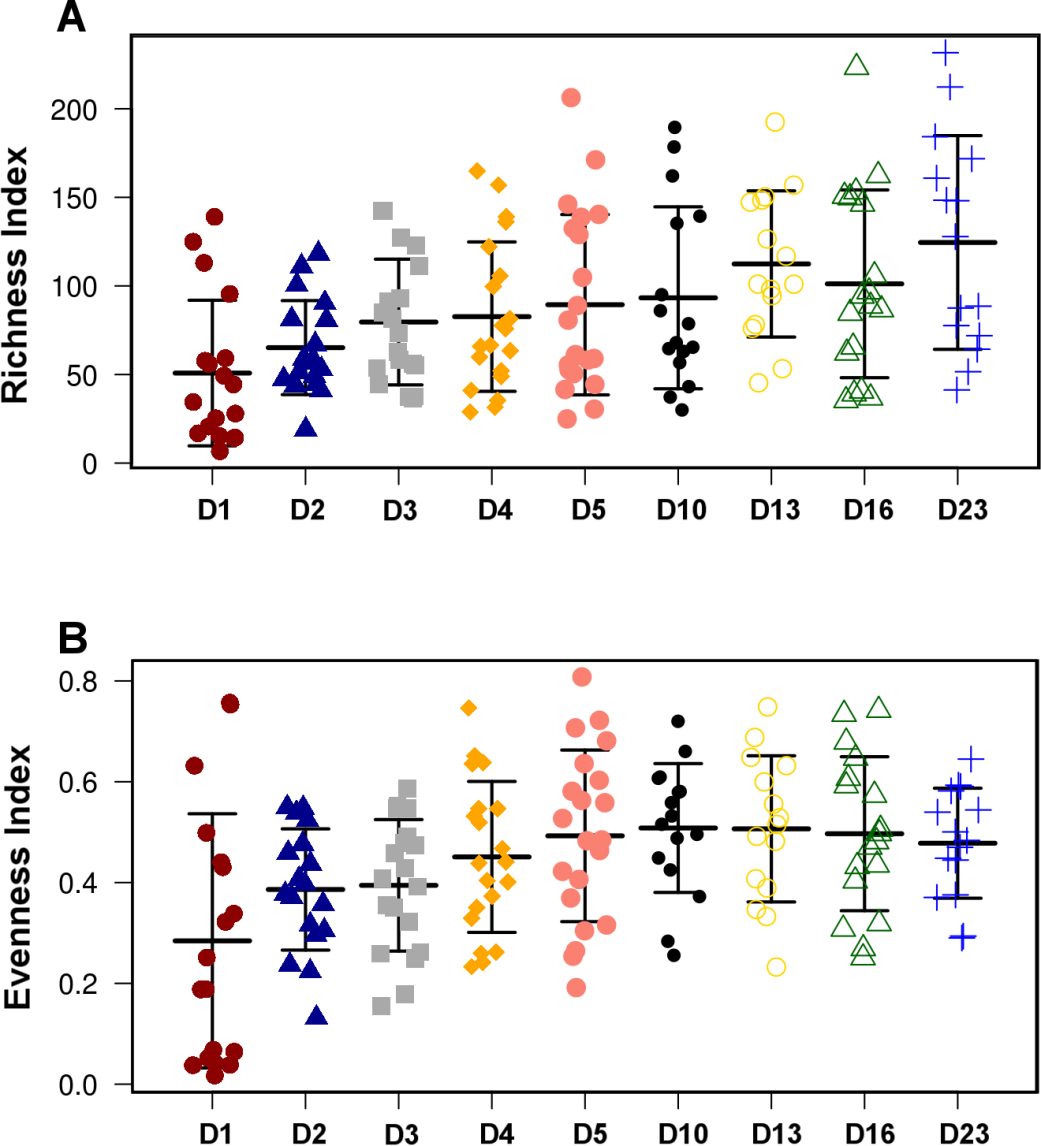
Overall diversity changes during the first 23 days of broiler microbiota development. Both Richness (A) and Evenness index (B) were significantly (ANOVA) different between the timepoints (*P* = 1.58*E*^−4^ and *P* = 2.02*E*^−4^, respectively). The present figure is composed of all three treatment groups, however, this trend of increasing richness and evenness reproducible in all individual treatment groups shown in Fig. S2.

### Microbial community development over time

The microbiota community analysis was extended to investigate the influence of treatments on microbiota development over time. Two-way PERMANOVA (Primer-E) was used on weighted and unweighted UniFrac distance matrices. On both weighted and unweighted UniFrac the influence of treatment was significant (*P* = 8.00*E*^−4^ and *P* < 1*E*^−5^, respectively) and differences between the days of community development were also significant by both weighted and unweighted UniFrac (both with *P* < 1*E*^−5^). The interaction between day vs. treatment was not significant by weighted (*P* = 0.225) and was significant by unweighted UniFrac (*P* = 0.011).

**Figure 3:**
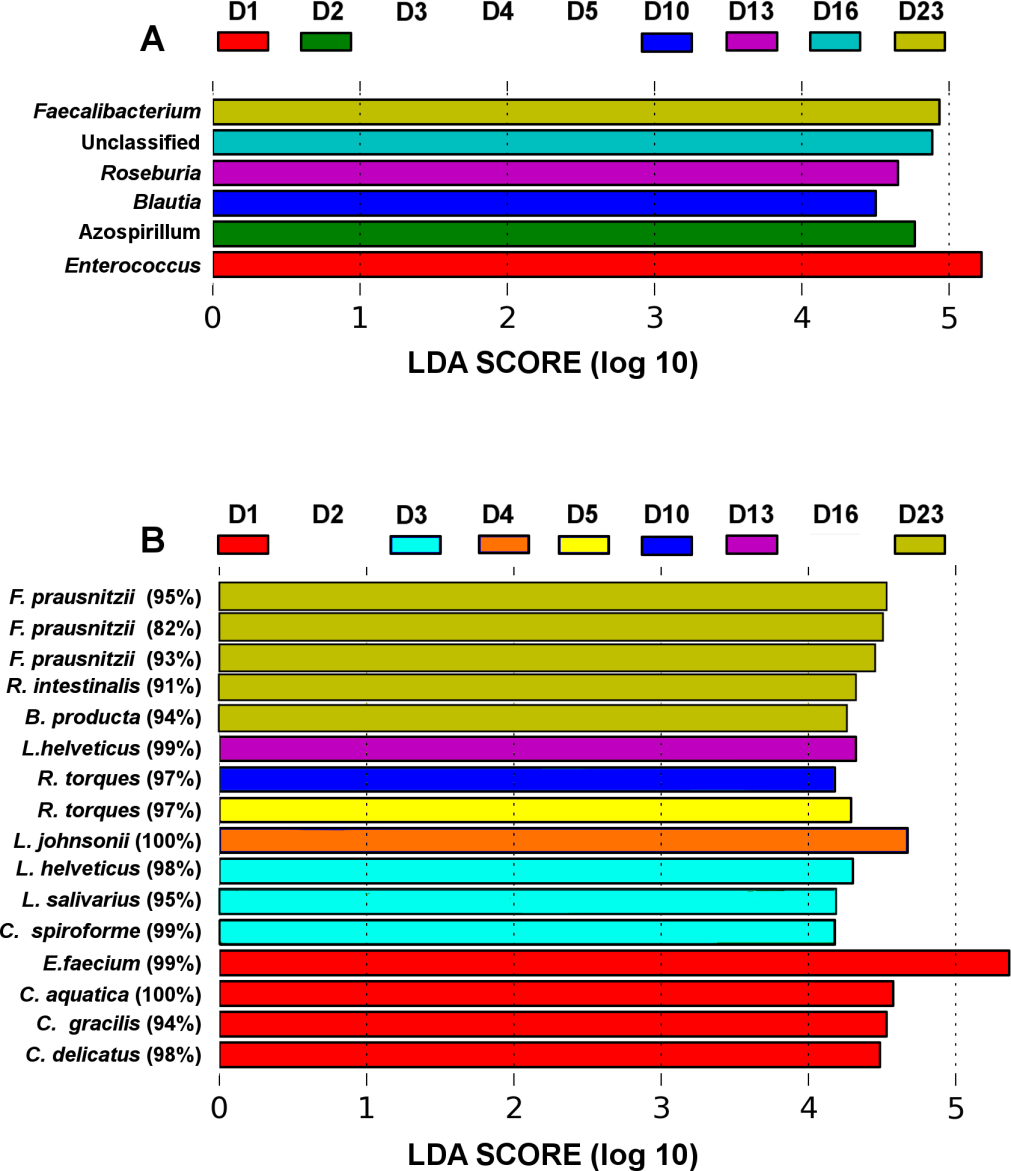
LEfSe analysis identified genera (A) and OTUs (B) that characterize the differences between the microbiota development stages. Only results from the top 50 most abundant genera and 100 most abundant OTUs are shown. OTUs are presented as their closest hit in NCBI *16S Microbial* database.

Although there were significant differences in microbial communities between the treatments, the community development timeline was comparable between the 3 groups. Overall species Richness and Evenness were altered significantly (ANOVA) during development of the microbial community (*P* = 1.58*E*^−4^ and *P* = 2.02*E*^−4^, respectively, Fig. 2) and the trend of increasing Richness and Evenness with time was reproducible across the three trials (Fig. S3). There was no significant difference in either Richness or Evenness between the three flocks.

The microbiota community development timelines were then compared in the three groups using LDA Effect Size (LEfSe) timeline analysis and classic Pearson regression to find taxa strongly associated with time. LEfSe analysis identified OTUs that characterize and drive the differences between two or more biological conditions (Segata et al., 2011). Major microbiota perturbations at the genus level (Fig. 3A) occurred at days 1 and 2 and again from days 10–23. *Enterococcus* and *Azospirillum* influenced the development differences at days 1 and 2 and *Blautia* and *Roseburia* from day 10 to 13 while an increase in abundance of *Faecalibacterium* was evident in more mature microbiota towards the end of the four week trial at day 23 (Fig. 3B).

**Figure 4:**
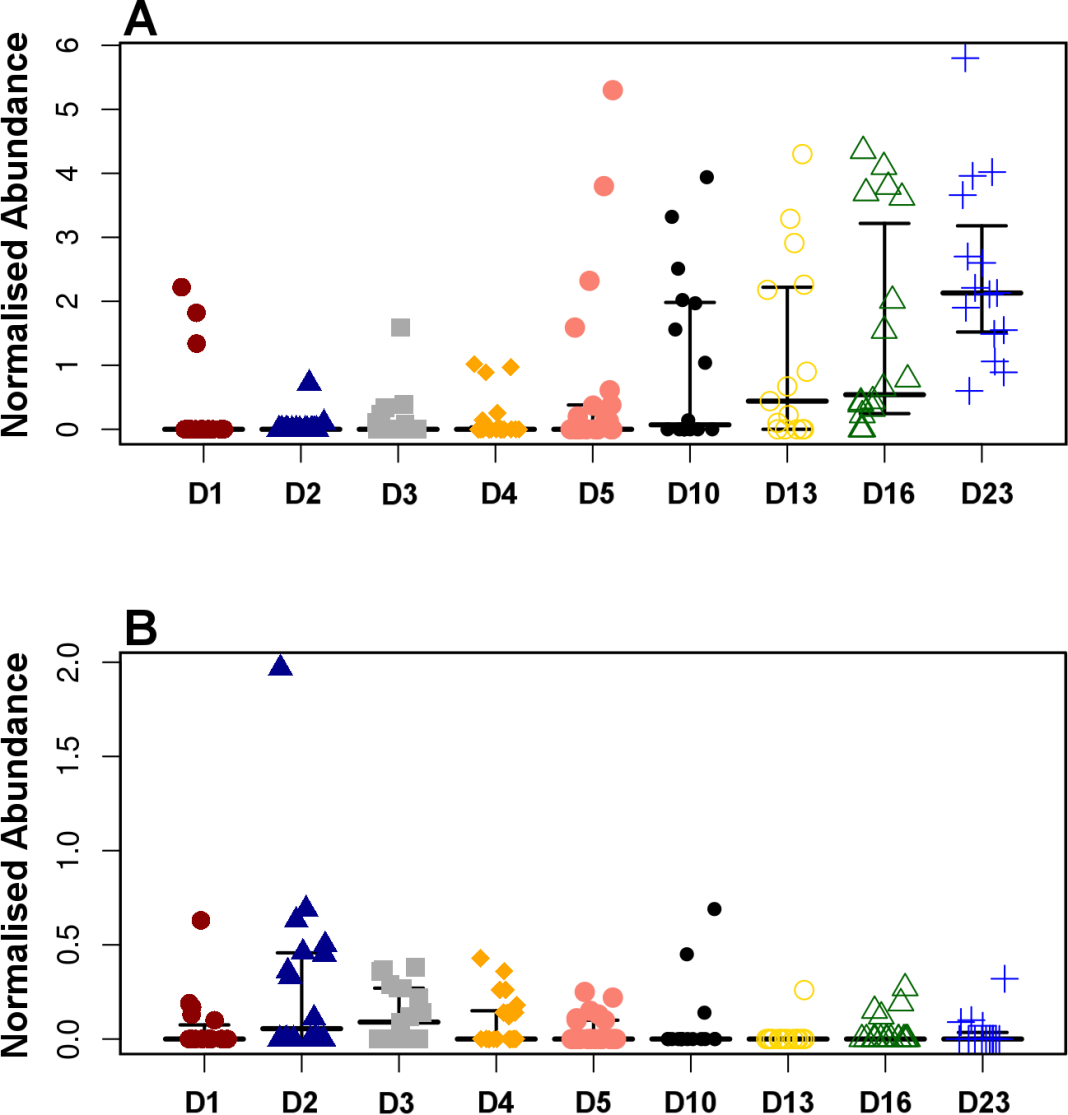
Development timeline for genera *Faecalibacterium* (A) and *Enterobacter* (B). *Faecalibacterium* was significantly positively correlated with timeline (*P* = 1.80*E*^−13^, *r* = 0.54) while *Enterobacter* was negatively correlated reducing with time (*P* = 9.8*E*^−3^, *r* =  − 0.20).

**Figure 5:**
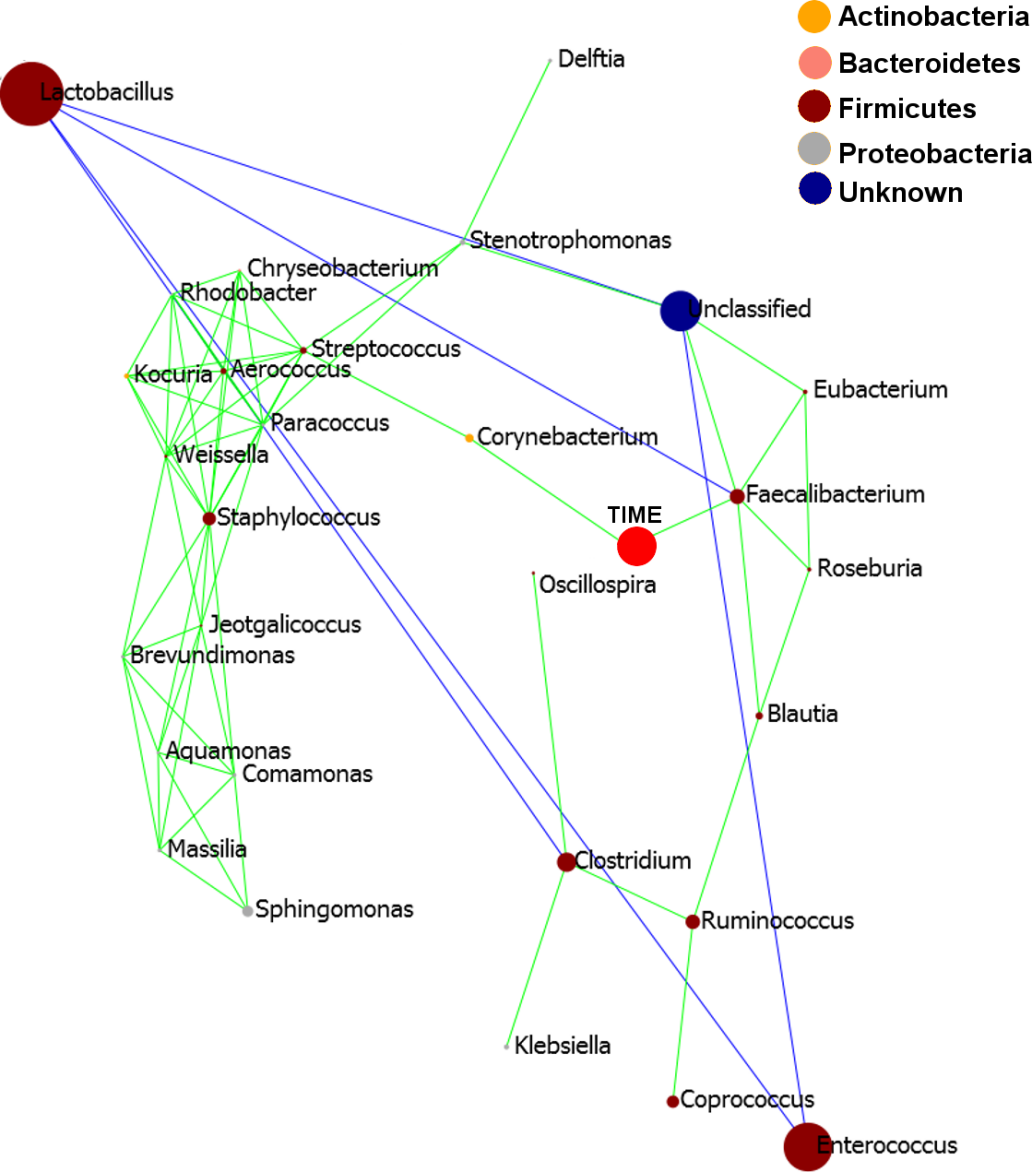
Pearson correlation network diagram showing interactions (Person correlations) between the 20 most abundant genera and time of development (Days). Positive Pearson correlations are presented in green and negative correlations in blue edges. The size of each node is proportional to the abundance of the genus, bigger circle indicating more abundant genera. The nodes representing each genus are coloured according to the phylum to which the genus belongs to and the legend provided.

Pearson based regression analysis was used to identify genera (Table 3) and OTUs (Table 4) significantly (*P* < 0.001) correlated with time (days). At the genus level *Faecalibacterium* steadily increased in abundance and was the most significantly positively correlated with time (*P* = 1.80*E*^−13^, *r* = 0.54; Table 3, Fig. 4A, Fig. S4), and was identified by LEfSe analysis as the genus that most distinguishes the later stages of microbiota development (Day 23). *Enterobacter* was significantly (*P* = 9.8*E*^−3^) negatively (*r* =  − 0.20) correlated with time (Table 3, Fig. 4B). *Enterococcus*, identified by LEftSe as driving differences in timeline during the first 2 days was not significantly correlated with time. Instead, it showed a reproducible pattern of colonisation by being very high during the first two days, diminished at day 3 and steadily increasing afterwards (Fig. S5). *Lactobacillus* did not change significantly during the time-course (Fig. S6). Interestingly, there were a number of genera that showed very little oscillation in abundance during the time-course and those include the least affected *Weissella* (*P* = 0.99). A network diagram visualising the genera interactions with one another during time of development is shown in Fig. 5. OTUs highly correlated with the timeline reflect the above mentioned time-responsive genera (Table 4).

**Table 3:** Pearson based correlations between the genera and time (days). Only genera with *P* < 0.01 are shown.

Genus	*P*-value	*R*	Present in samples (of 160)
*Faecalibacterium*	1.80E–13	0.5395	73
*Proteus*	4.80E–06	0.3526	11
*Corynebacterium*	4.40E–05	0.3170	63
*Anaerotruncus*	1.50E–04	0.2948	15
Unclassified	1.50E–04	0.2950	160
*Oscillospira*	2.50E–04	0.2854	53
*Blautia*	5.50E–04	0.2703	106
*Acinetobacter*	9.60E–04	−0.2586	36
*Klebsiella*	0.0011	−0.2558	59
*Pseudomonas*	0.0011	−0.2562	31
*Comamonas*	0.0020	−0.2430	108
*Flavobacterium*	0.0037	−0.2284	52
*Abiotrophia*	0.0038	0.2278	7
*Raoultella*	0.0040	−0.2262	50
*Escherichia*	0.0043	−0.2247	48
*Enterobacter*	0.0098	−0.2038	52

**Table 4:** Pearson based correlations between the OTUs and time (days). The blastn hit against *16S Microbial* NCBI database with highest % identity (%ID). Only OTUs with *P* < 0.001 and |*r*| > 0.4 are shown.

OTU ID	*P*	*R*	Present in samples (of 160)	Blast hit (16S NCBI database)	%ID
71,659	2.2E^−13^	0.53	71	*Faecalibacterium prausnitzii* ATCC 27768	95
98,307	1.7E^−12^	0.52	29	*Roseburia intestinalis* L1-82	91
81,371	2.4E^−12^	0.52	30	*Enterococcus casseliflavus* EC20	93
26,571	5.2E^−12^	0.51	22	*Faecalibacterium prausnitzii* ATCC 27768	89
93,200	8.5E^−12^	0.51	17	*Enterococcus casseliflavus* EC20	90
37,945	9E^−12^	0.50	21	*Enterococcus casseliflavus* EC20	93
103,950	1E^−11^	0.50	28	*Ruminococcus torques* VPI B2-51	96
68,588	1.6E^−11^	0.50	22	*Enterococcus casseliflavus* EC20	89
14,568	5.2E^−11^	0.50	53	*Faecalibacterium prausnitzii* ATCC 27768	87
101,095	5.4E^−11^	0.49	14	*Faecalibacterium prausnitzii* ATCC 27,768	88
4,265	5.7E^−11^	0.49	23	*Faecalibacterium prausnitzii* ATCC 27768	86
96,474	2.1E^−10^	0.47	14	*Faecalibacterium prausnitzii* ATCC 27768	91
51,606	3.3E^−10^	0.47	56	*Faecalibacterium prausnitzii* ATCC 27768	93
92,572	6.6E^−10^	0.46	12	*Faecalibacterium prausnitzii* ATCC 27768	90
82,276	1E^−09^	0.46	135	*Enterococcus casseliflavus* EC20	99
47,882	1.3E^−09^	0.46	12	*Faecalibacterium prausnitzii* ATCC 27768	92
114,950	1.4E^−09^	0.46	26	*Faecalibacterium prausnitzii* ATCC 27768	82
93,380	1.5E^−09^	0.45	34	*Subdoligranulum variabile* BI 114	94
118,252	3.3E^−09^	0.45	12	*Enterococcus casseliflavus* EC20	85
72,184	3.5E^−09^	0.45	34	*Oscillibacter valericigenes* Sjm18-20	83
80,030	3.6E^−09^	0.44	28	*Enterococcus casseliflavus* EC20	96
54,054	3.7E^−09^	0.44	24	*Faecalibacterium prausnitzii* ATCC 27768	94
18,075	5.7E^−09^	0.44	16	*Roseburia hominis* A2-183	96
18,512	5.7E^−09^	0.44	16	*Faecalibacterium prausnitzii* ATCC 27768	91
96,693	6.5E^−09^	0.44	55	*Ruminococcus torques* VPI B2-51	94
9,187	6.8E^−09^	0.45	17	*Faecalibacterium prausnitzii* ATCC 27768	93
76,037	7.3E^−09^	0.44	15	*Faecalibacterium prausnitzii* ATCC 27768	93
65,998	8.8E^−09^	0.43	18	*Lactobacillus crispatus* ST1	98
106,613	8.9E^−09^	0.43	35	*Faecalibacterium prausnitzii* ATCC 27768	97
121,284	9E^−09^	0.43	68	*Roseburia intestinalis* L1-82	92
35,061	9.6E^−09^	0.43	31	*Acetanaerobacterium elongatum* Z7	79
94,779	1.1E^−08^	0.43	42	*Enterococcus casseliflavus* EC20	97
28,314	1.1E^−08^	0.43	17	*Enterococcus casseliflavus* EC20	86
34,854	1.3E^−08^	0.43	22	*Ruminococcus champanellensis* 18P13	81
117,006	1.3E^−08^	0.43	14	*Enterococcus casseliflavus* EC20	91
99,064	1.4E^−08^	0.43	25	*Acetanaerobacterium elongatum* Z7	84
10,778	2.6E^−08^	0.42	12	*Acetanaerobacterium elongatum* Z7	79
99,661	2.6E^−08^	0.42	26	*Lactobacillus crispatus* ST1	91
13,781	2.8E^−08^	0.42	14	*Faecalibacterium prausnitzii* ATCC 27768	89
77,207	4E^−08^	0.42	41	*Enterococcus casseliflavus* EC20	96
82,833	7.3E^−08^	0.41	25	*Eubacterium tortuosum*	88
68,948	7.4E^−08^	0.41	19	*Enterococcus casseliflavus* EC20	94
46,046	7.7E^−08^	0.41	17	*Streptococcus uberis* 0140J	79
83,461	1.1E^−07^	0.40	22	*Subdoligranulum variabile* BI 114	93
23,303	1.2E^−07^	0.40	23	*Enterococcus casseliflavus* EC20	87
96,525	1.3E^−07^	0.40	42	*Enterococcus casseliflavus* EC20	98
68,889	1.3E^−07^	0.40	62	*Enterococcus casseliflavus* EC20	99
102,592	1.1E^−09^	−0.46	105	*Enterococcus faecium* Aus0004	99

## Discussion

This investigation aimed to determine whether treatment of eggs with cecal microbiota could improve the uniformity of microbiota development in the growing chicks and reduce bird-to-bird variation. The results indicated that one of the cecal treatments did achieve higher uniformity and reduced bird-to-bird microbiota variation significantly using both weighted and unweighted UniFrac; however, this was not reproduced in the other cecal treatment using an inoculum with a different microbiota profile. High bird-to-bird variations were not completely removed in either cecal treatment. However, it is possible that cecal treatment could prove beneficial by preventing formation of atypical communities which we have encountered in some flocks and discussed previously (Stanley et al., 2013c). Considering that both treatments were prepared using the same methodology but contained different cecal microbiota inocula, originating from different birds, this inconsistency could be partially explained by differences in resistance to freezing of the cecal inoculum and the difference in the ability to colonise between the two inoculum communities.

It has been reported previously that bacteria subjected to this freezing/thawing process may be damaged and can display alterations in the lag phase and in growth rate (Postgate & Hunter, 1961), however, preparing fresh inoculum for each fecal/cecal transfer is impractical in industry and clinical practice alike, and despite the loss of cold sensitive microbiota, frozen bacterial communities do not show significant differences in fecal tranfer success compred to the fresh sample (Lee et al., 2016). However, oxygen in the atmosphere, gastric acid, bile salts and harsh upper GIT environment do play a major role in colonisation efficiency. We used swabbing of eggs with fecal transfer as a mimic of natural microbiota transfer from adult birds and nesting material and as a practical microbiota transfer method. Total anaerobic preparation of inoculum and anaerobic methods of inoculation were discounted for practical reasons and their unlikely use in hatcheries. Therefore, it was expected that strictly anaerobic bacteria would not survive the treatment and hence would not be transferred, and that is indeed what was found with no transfer of the strictly anaerobic *Bacteroides* that were a dominant population in inoculum 2. It had been hoped that the other community members of the microbiota in inoculum 2 may create an environment suitable for proliferation of *Bacteroides* species acquired by other routes but this did not seem to be the case.

Understanding the dynamics and interactions during formation of intestinal microbial communities is of high relevance for microbiota modifications. Overall, only a few genera significantly correlated with the time post-hatch and during the period of gut microbiota maturation. *Enterococcus* were the most abundant species in the day one community, followed by *Lactobacillus* from day two which showed a high level of variation in subsequent samplings. *Faecalibacterium* is a genus known to be associated with more mature microbiota in humans (Rodriguez et al., 2015), consistent with our findings.

The abundance of *Enterobacter* in the chicken GIT was found to be negatively correlated with time (as the chickens aged). The early colonisation in newly hatched chicks, as well as human infants, can be explained by the initial environment of the GIT. Enterobacteriaceae, the family to which this genus belongs, and *Lactobacillus*, are both well-known early colonisers of the GIT. Their facultative anaerobic properties enable them to utilise the initial oxygen supplies in the gut during the first days of life. The subsequent depletion of this oxygen supply then creates a more favourable environment for obligate anaerobes to develop and thrive (Matamoros et al., 2013). The reduction in Enterobacteriaceae numbers over time can be influenced by the production of short chain fatty acids (SCFA) in the cecum of the chickens. SCFA reduce the intestinal pH and have an inhibitory effect on bacteria that are considered acid-sensitive, such as Enterobacteriaceae. This mechanism assists in the prevention of overgrowth and the potential pathogenicity that is associated with high levels of Enterobacteriaceae in the GIT. Furthermore, this reduction in abundance is consistent with studies that suggest the family Enterobacteriaceae is ideally present in only small amounts in the gut of healthy adult humans (Candela et al., 2012; Collado et al., 2005; Shin, Whon & Bae, 2015).

*Faecalibacterium* is an obligate anaerobe and thus typically does not proliferate and colonise until such time as a favourable intestinal environment has been set. In the healthy human adult, *Faecalibacterium* becomes so abundant that they have been found to represent more than 5% of the microbiota population in some cases. Furthermore, studies have indicated that a lack of this bacteria in the human adult gut is associated with inflammatory bowel diseases. Equally, its abundance has the potential to be used as a biomarker for human intestinal health (Benus et al., 2010; Sokol et al., 2009). It is expected that the same is true in chickens and *Faecalibacterium* is likely to strongly influence their immune development.

## Conclusions

The dynamics of gut microbiota development in chickens, over time, has been demonstrated as highly reproducible in the three treatment groups with distinct microbiota and the development was independent from treatment differences, as shown by PERMANOVA analysis. This knowledge of developmental timelines may be used in the future to guide strategies for microbiota manipulation, culturing efforts and timing of probiotic treatments.

##  Supplemental Information

10.7717/peerj.3587/supp-1 Supplemental Information 1 Figures S1-S6

## Additional Information and Declarations

### Data Availability

The following information was supplied regarding data availability: The sequencing data is publically available on MG-RAST database under library number mgl564762 and project ID mgp21821.
