# Supplementary material for: The time-course of broiler intestinal microbiota development after administration of cecal contents to incubating eggs
Source: PeerJ. 2017 Jul 20;5:e3587. doi: 10.7717/peerj.3587 (PMC5522604; doi:10.7717/peerj.3587)
Supplement: Supplemental Information 1 [file peerj-05-3587-s001.pdf]

The time-course of broiler intestinal microbiota development after administration of cecal contents to incubating eggs

Erin Donaldson[1], Dragana Stanley[1,2], Robert J. Hughes[2,3,4], Robert J. Moore[2,5]

[1]Institute for Future Farming Systems, Central Queensland University, Rockhampton, Queensland, Australia

[2]Poultry Cooperative Research Centre, University of New England, Armidale, New South Wales, Australia

[3]South Australian Research and Development Institute, Pig and Poultry Production Institute, Roseworthy, South Australia 5371, Australia

[4]The University of Adelaide, School of Animal and Veterinary Sciences Roseworthy, South Australia 5371, Australia

[5]RMIT University, School of Science, Bundoora, Victoria 3083, Australia

[§]Corresponding author

Robert Moore

RMIT University, Bundoora West Campus

Bundoora Vic. 3083 Australia

Email: rob.moore@rmit.edu.au

Phone: +61 3 9925 7580

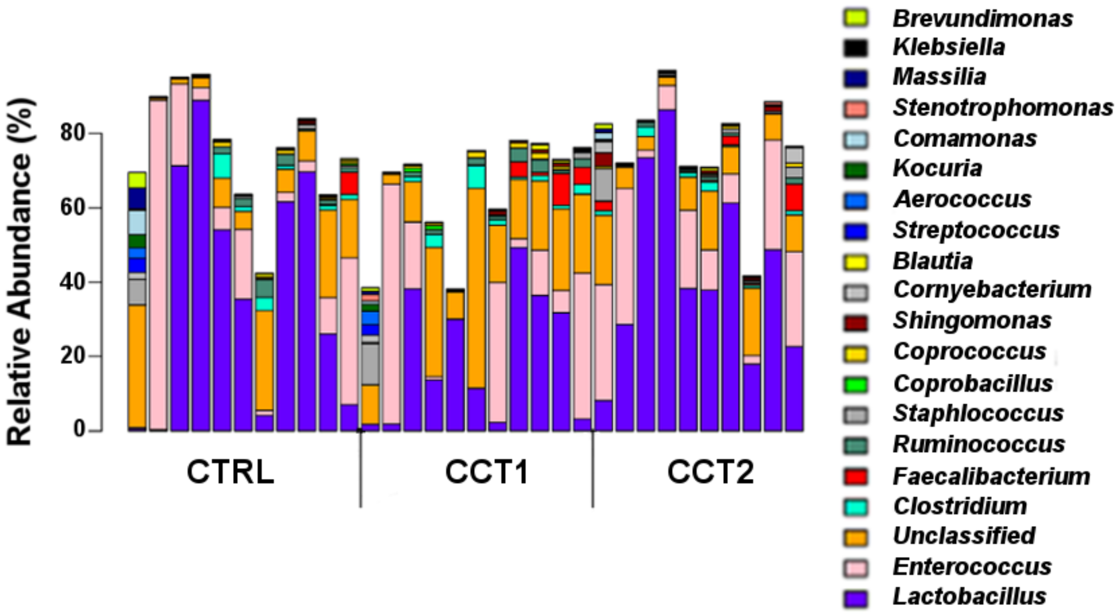

Figure S1: Top 20 genera distribution across the treatments

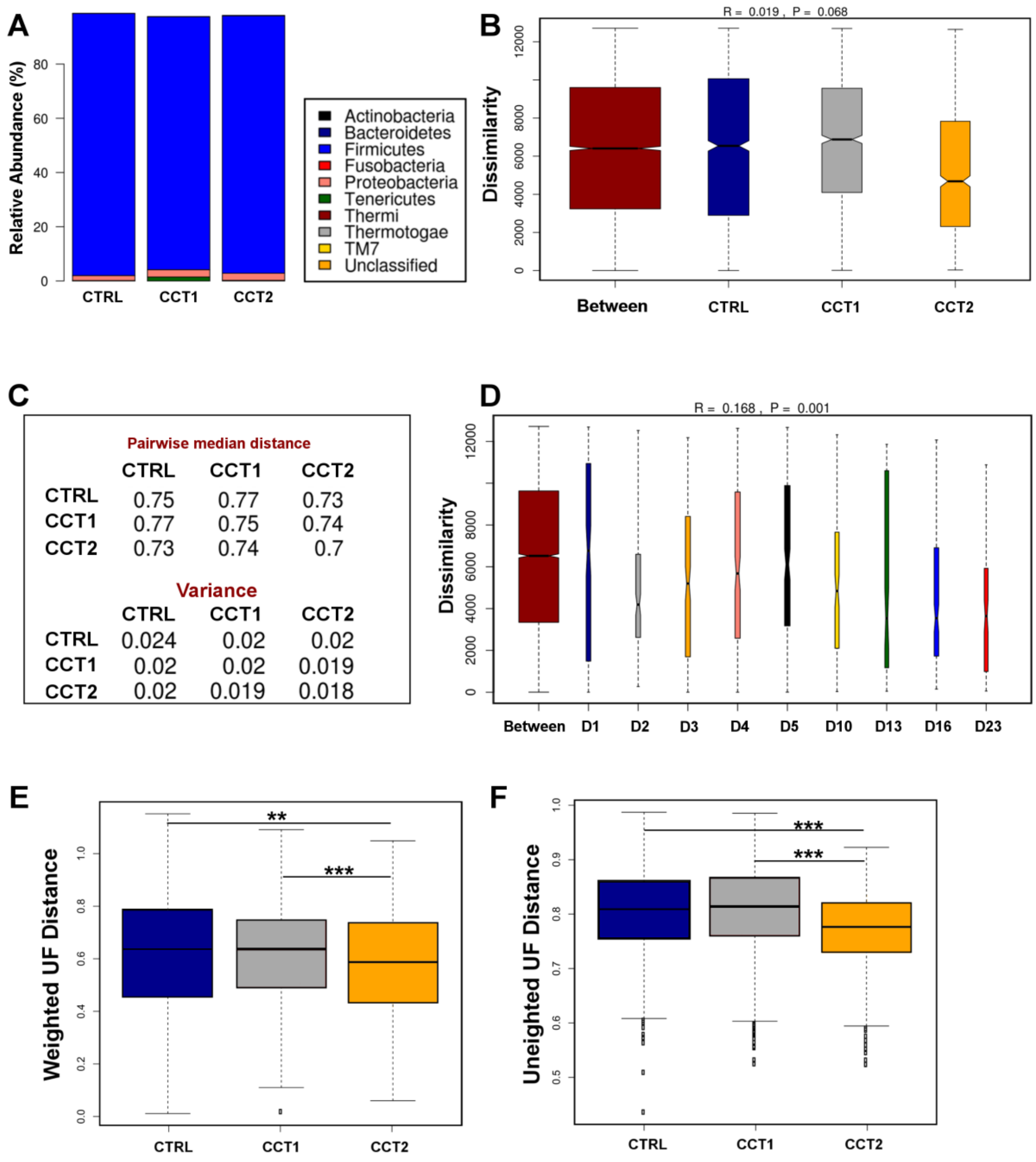

Figure S2. Mean phylum abundance distribution across the 3 flocks (A); notched boxplot representation of ANOSIM analysis (B) of treatment groups with dissimilarity based on Bray–Curtis distance. Sample dissimilarity within each group is indicated in the graph and overall dissimilarity is indicated as “Between”. Width of the boxplot is proportional to number of samples in each treatment; Pearson inter and intra-group sample correlations and group variance (C) and ANOSIM representation of the days of microbiota development (D). UniFrac between samples distances were different between the groups in both weighted and unweighted UniFrac (E and F).

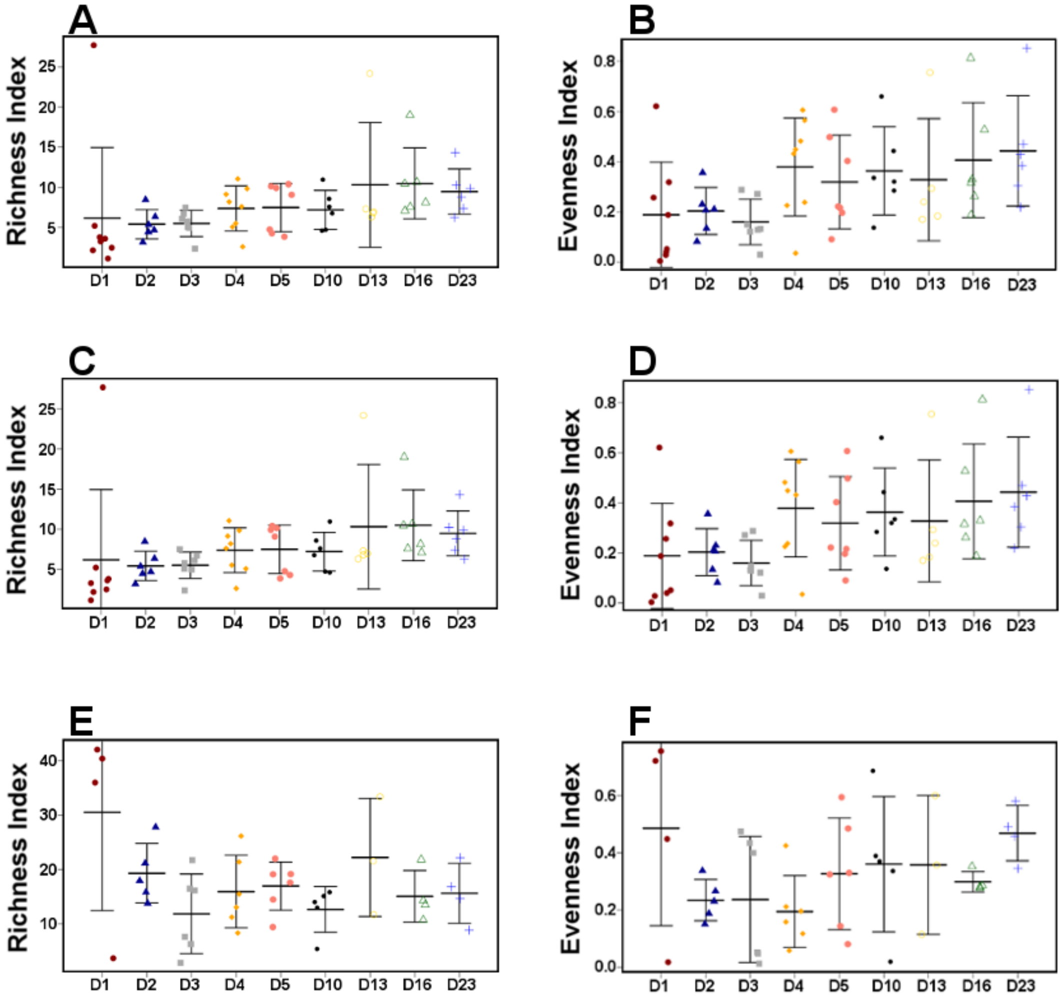

Figure S3: Richness and evenness index reproducibly increased in all 3 flocks. Control flock is shown in panels A and B, CCT1 in C and D and CCT2 in E and F.

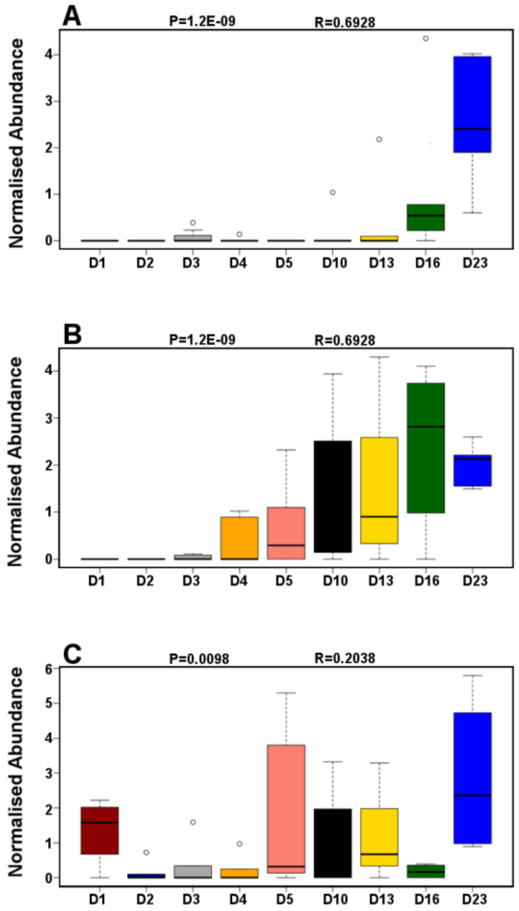

Figure S4: Changes in abundance of genus *Faecalibacterium* in cloacal samples in the control group (A), CCT1 (B) and CCT2 (B) as chickens aged. *Faecalibacterium* was significantly correlated with age in all 3 groups.

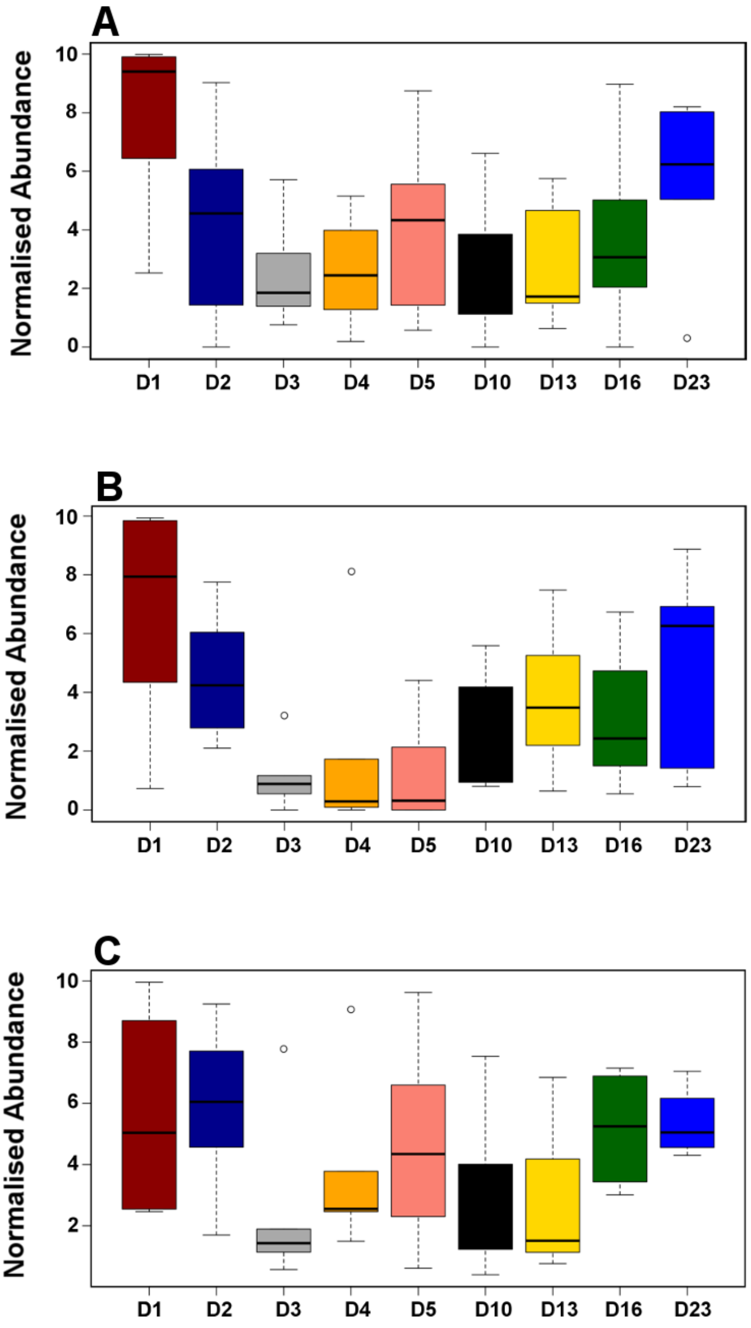

Figure S5: Changes in abundance of genus *Enterococcus* in cloacal samples in the control group (A), CCT1 (B) and CCT2 (B) as chickens aged. LEfSe analysis indicated *Enterococcus* as defining timeline genera abundance differences at day 1 and 2. *Enterococcus* showed fluctuations in the day 1 and 2 and then steady growth afterwards but did not significantly correlate with time.

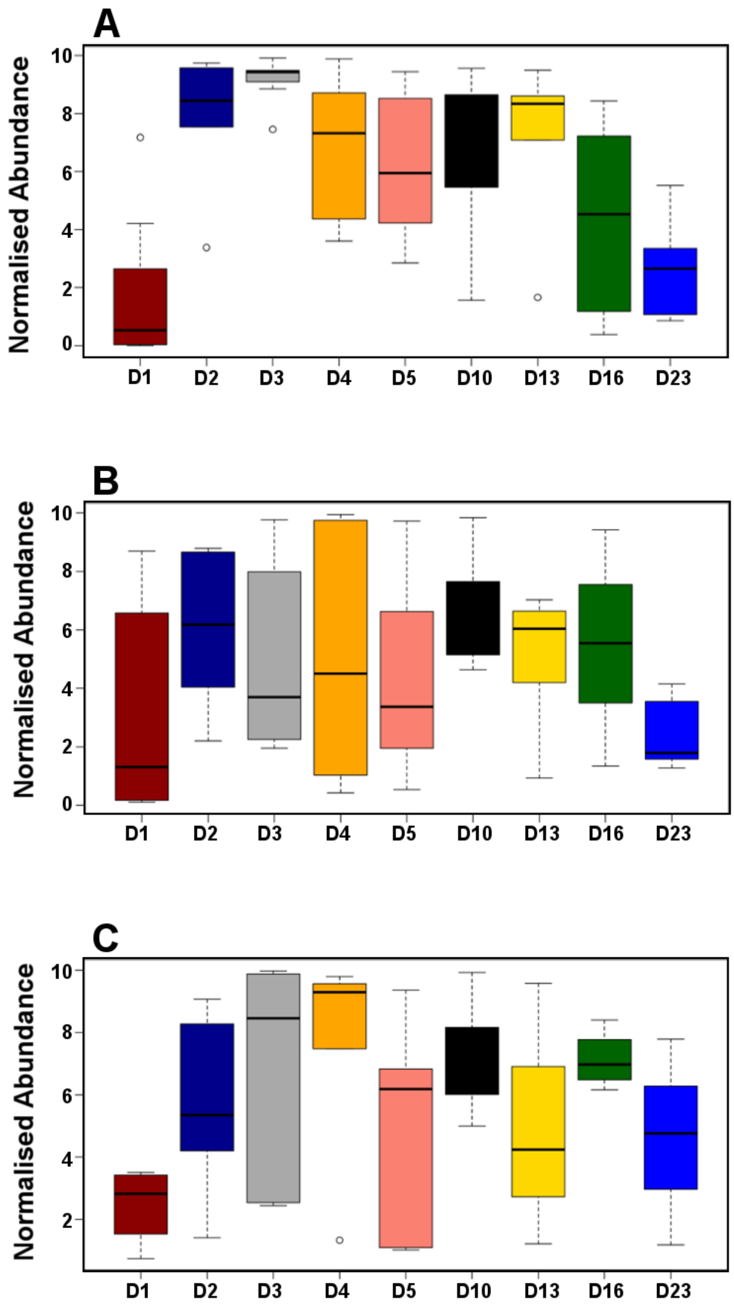

Figure S6: Changes in abundance of genus *Lactobacillus* in cloacal samples in the control group (A), CCT1 (B) and CCT2 (B) as chickens aged. There were no significant correlations with time.
